# Supplementary figures and images for: A novel genus of virulent phage targeting Acinetobacter baumannii: Efficacy and safety in a murine model of pulmonary infection
Source: PLoS Pathog. 2025 Jun 20;21(6):e1013268. doi: 10.1371/journal.ppat.1013268 (PMC12208414; doi:10.1371/journal.ppat.1013268)

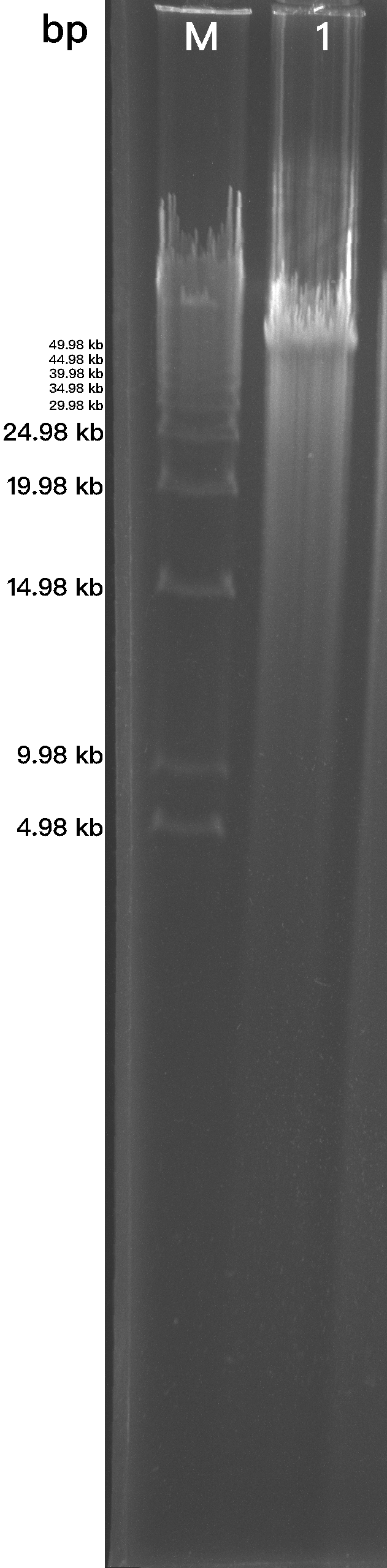

Supplement: S1 Fig — M: DNA Size Standards - 5 kb ladder (BIO-RAD); 1: vB_AbaS_qsb1 genome. (TIF) [file ppat.1013268.s001.tif]

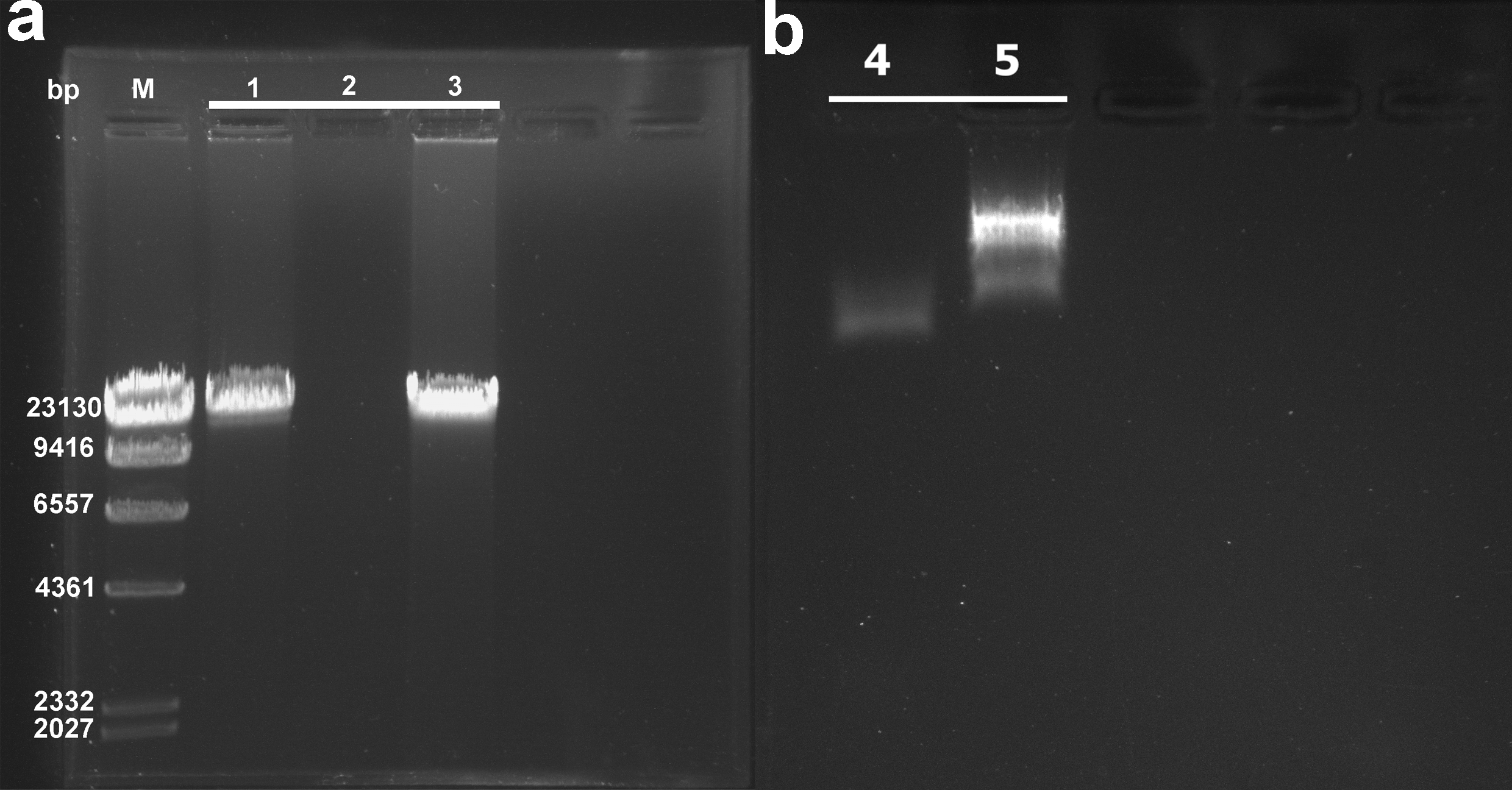

Supplement: S2 Fig — (TIF) [file ppat.1013268.s002.tif]

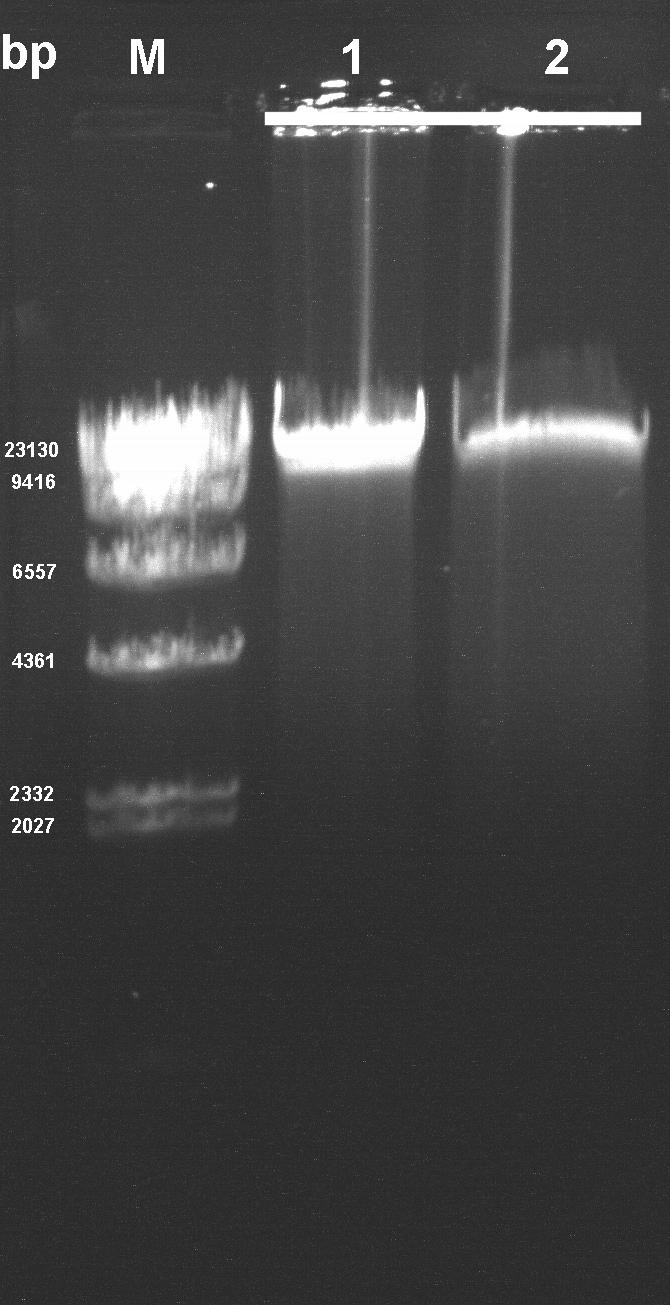

Supplement: S3 Fig — (TIF) [file ppat.1013268.s003.tif]
