# Supplementary material for: A novel genus of virulent phage targeting Acinetobacter baumannii: Efficacy and safety in a murine model of pulmonary infection
Source: PLoS Pathog. 2025 Jun 20;21(6):e1013268. doi: 10.1371/journal.ppat.1013268 (PMC12208414; doi:10.1371/journal.ppat.1013268)
Supplement: S1 Methods — (DOCX) [file ppat.1013268.s009.docx]

**Supplementary methods: for analysis of bacterial and phage colonization, inflammatory cytokine levels, and histopathological examination in mice**

**Determination of bacterial and phage colonization**

After the mice were sacrificed, their lungs were aseptically removed, weighed, and mechanically homogenized in PBS containing protease inhibitors (PMSF). The lung homogenates were kept on ice and serially diluted within 1 hour. The bacterial load (CFU) in the lungs was quantified using standard microbiological methods, while the phage titer (PFU) of bacteriophage qsb1 was determined using the DLA method. Finally, the unit is CFU/g or PFU/g.

**Cytokine quantification**

For the treated lung tissue homogenate, the concentrations of interleukins (IL-1β, IL-6) and tumor necrosis factor-α (TNF-α) were quantified using ELISA. Cytokine levels were expressed the total protein relative content in the lung homogenate, measured as the optical density at 450 nm (OD_450nm_).

**Histopathological Analysis**

Following euthanasia, lung tissues were harvested and immediately fixed in 4% paraformaldehyde. The fixed tissues were processed for dehydration using an automated tissue dehydration processor (HistoCore PEGASUS, Leica), embedded in paraffin (HistoCore Arcadia H&C, Leica), and sectioned into 4 μm thick slices using a microtome (HistoCore Multicut, Leica). Sections were stained with hematoxylin and eosin (H&E) utilizing an automated staining system (HistoCore CHROMAX ST, Leica) and coverslipped with an automated coverslipper (Leica CV5030). Digital images of the stained sections were acquired and analyzed using a digital pathology slide scanner (KF-PRO-005-EX, KFBIO).
