# Supplementary material for: A novel genus of virulent phage targeting Acinetobacter baumannii: Efficacy and safety in a murine model of pulmonary infection
Source: PLoS Pathog. 2025 Jun 20;21(6):e1013268. doi: 10.1371/journal.ppat.1013268 (PMC12208414; doi:10.1371/journal.ppat.1013268)
Supplement: Attachment — Submitted filename: Response to Reviewers.docx [file ppat.1013268.s011.docx]

­­­PLOS Pathogens

Ref: PPATHOGENS-D-24-02438­­­

A Novel Genus of Virulent Phage Targeting *Acinetobacter baumannii*: Efficacy and Safety in a Murine Model of Pulmonary Infection
Response letter to Reviewers’ comments

Dear Editors and reviewers:

Thank you very much for considering our manuscript to be published in **PLOS Pathogens**. We have revised our manuscript according to the reviewers’ comments. All revised contents have been highlighted by yellow color in the revised manuscript. Furthermore, the point-to-point responses have also been presented in this response letter.

We hope this revision will meet the high standard of PLOS Pathogens.

**Response to the Editors:**

1) We do not publish any copyright or trademark symbols that usually accompany proprietary names, eg ©, ®, or TM (e.g. next to drug or reagent names). Therefore please remove all instances of trademark/copyright symbols throughout the text, including:

-TM on page: 26 line 502.

**Response: Thank you for your valuable comments, which are very meaningful for us to further improve our manuscript. As you said, in order to comply with the relevant requirements for publishing articles in your journal, we have deleted all trademark/copyright symbols in the article.**

**Original content: Illumina NovaSeq^TM^ X Plus system**

**Current content: Illumina NovaSeq X Plus system——This sentence now appears at L630 in the revised manuscript.**

2) We have noticed that you have uploaded Supporting Information files, but you have not included a list of legends. Please add a full list of legends for your Supporting Information files after the references list.

**Response: Thank you for pointing out the problem with the manuscript. We have now added a full list of legends information of the supporting information to the manuscript. ——This sentence now appears at L733-L745 in the revised manuscript.**

3) Some material included in your submission may be copyrighted. According to PLOSu2019s copyright policy, authors who use figures or other material (e.g., graphics, clipart, maps) from another author or copyright holder must demonstrate or obtain permission to publish this material under the Creative Commons Attribution 4.0 International (CC BY 4.0) License used by PLOS journals. Please closely review the details of PLOSu2019s copyright requirements here: PLOS Licenses and Copyright. If you need to request permissions from a copyright holder, you may use PLOS's Copyright Content Permission form.

Please respond directly to this email and provide any known details concerning your material's license terms and permissions required for reuse, even if you have not yet obtained copyright permissions or are unsure of your material's copyright compatibility. Once you have responded and addressed all other outstanding technical requirements, you may resubmit your manuscript within Editorial Manager.

Potential Copyright Issues:

i) Please confirm (a) that you are the photographer of 1A, or (b) provide written permission from the photographer to publish the photo(s) under our CC BY 4.0 license.

**Response: (a) that you are the photographer of 1A.**

ii) Graphical Abstract. Please confirm whether you drew the images / clip-art within the figure panels by hand. If you did not draw the images, please provide (a) a link to the source of the images or icons and their license / terms of use; or (b) written permission from the copyright holder to publish the images or icons under our CC BY 4.0 license. Alternatively, you may replace the images with open source alternatives. See these open source resources you may use to replace images / clip-art:

- https://commons.wikimedia.org

- https://openclipart.org/.

**Response:** **Yes, the graphical abstract was hand-drawn by author of the manuscript. Likewise, we also store the original SVG line artwork for the images.**

4) In the online submission form, you indicated that "The datasets generated during and/or analyzed during the current study are available from the corresponding author on reasonable request." All PLOS journals now require all data underlying the findings described in their manuscript to be freely available to other researchers, either

1. In a public repository

2. Within the manuscript itself

3. Uploaded as supplementary information.

This policy applies to all data except where public deposition would breach compliance with the protocol approved by your research ethics board. If your data cannot be made publicly available for ethical or legal reasons (e.g., public availability would compromise patient privacy), please explain your reasons by return email and your exemption request will be escalated to the editor for approval. Your exemption request will be handled independently and will not hold up the peer review process, but will need to be resolved should your manuscript be accepted for publication. One of the Editorial team will then be in touch if there are any issues.

**Response: Thank you for your valuable comments, which are very meaningful for us to further improve our manuscript. The raw data information related to this study has been uploaded to the dataset figshare (https://figshare.com/), with the DOI number “10.6084/m9.figshare.27635490”**(https://figshare.com/articles/dataset/My_rawdata/27635490?file=52193141)**. These raw data were uploaded to the dataset when the manuscript was first submitted, but it may be due to technical reasons that we forgot to set it to be publicly visible, resulting in the editors and reviewers being unable to see our raw data. This error has now been corrected, and the raw data can be browsed or downloaded online.——L657**

5) Please amend your detailed Financial Disclosure statement. This is published with the article. It must therefore be completed in full sentences and contain the exact wording you wish to be published.

1) State the initials, alongside each funding source, of each author to receive each grant. For example: "This work was supported by the National Institutes of Health (####### to AM; ###### to CJ) and the National Science Foundation (###### to AM)."
**Response: Thank you for your valuable comments, which are very meaningful for us to further improve our manuscript. As you said,** **we already put the initials of each author who received each grant next to each funding source. ——This sentence now appears at L750-L756 in the revised manuscript.**

**Original content:** This work was supported by the Fujian Provincial Department of Science and Technology (No. 2024J011223); Fuzhou Municipal Health Commission (No. 2022-S-603 wr4) and (No. 2022-S-rc5); Ministry of Science and Technology of the People's Republic of China (No. 2023YFC3304304); Fuzhou University (No. 2024T021);Fujian Provincial Department of Science and Technology (No. 2022Y4003) and (No.2022ZD01001);

**Current content:** This work was supported by the Fujian Provincial Department of Science and Technology (No. 2024J011223 to XLY ); Fuzhou Municipal Health Commission (No. 2022-S-wr4 to XLY) and (No. 2022-S-rc5 to XLY); Ministry of Science and Technology of the People's Republic of China (No. 2023YFC3304304 to JL); Fuzhou University (No. 2024T021 to HH); Fujian Provincial Department of Science and Technology (No. 2022Y4003 to JL); Fujian Provincial Major Health Research Project (No. 2022ZD01001 to JL); The funders had no role in study design, data collection and analysis, decision to publish, or preparation of the manuscript.——L644-650

2) State what role the funders took in the study. If the funders had no role in your study, please state: "The funders had no role in study design, data collection and analysis, decision to publish, or preparation of the manuscript.".

**Response: Thank you for your valuable comments, which are very meaningful for us to further improve our manuscript. Our funders were not involved in our research process. Therefore, we added "The funders had no role in study design, data collection and analysis, decision to publish, or preparation of the manuscript" to the funding disclosure section. ——L644-650**

1) Thank you for stating "The bacteriophage strain vB_AbaS_qsb1 has been filed for a patent."

Please include a completed 'Competing Interests' statement in the text box when submitting your production task, including any COIs declared by your co-authors, written in full sentences.

If you have competing interests, please ensure the statement is in full sentences and includes the beginning statement: "I have read the journal's policy and the authors of this manuscript have the following competing interests:

NOTE: If you have no competing interests to declare, please state "The authors have declared that no competing interests exist". You may also provide an updated statement via email.

**Response: Thank you for your valuable comments, which are very meaningful for us to further improve our manuscript.** **We have updated the Competing Interests statement in the submission system. The statement now follows the required format.**

2) Please ensure that the affiliations of the authors listed on the manuscript title page do exactly match with the affiliations provided in the online submission form

NOTE: Affiliations should include a department (if applicable), an institution, a CITY, and a country

**Response: Thank you for your valuable comments, which are very meaningful for us to further improve our manuscript. We have ensured that the affiliations in the manuscript title page exactly match those in the submission form, including department, institution, city, and country.**

3) In the online submission form, you indicated that The datasets generated during and/or analyzed during the current study are available from the corresponding author on reasonable request. All PLOS journals now require all data underlying the findings described in their manuscript to be freely available to other researchers, either

1. In a public repository

2. Within the manuscript itself

3. Uploaded as supplementary information.

This policy applies to all data except where public deposition would breach compliance with the protocol approved by your research ethics board. If your data cannot be made publicly available for ethical or legal reasons (e.g., public availability would compromise patient privacy), please explain your reasons by return email and your exemption request will be escalated to the editor for approval. Your exemption request will be handled independently and will not hold up the peer review process, but will need to be resolved should your manuscript be accepted for publication. One of the Editorial team will then be in touch if there are any issues.

**Response: Thank you for your valuable comments, which are very meaningful for us to further improve our manuscript. We have revised the data availability statement, stating that all our raw data are now stored in the public database Figshare**

4) In the online submission form you indicate that your data is available from a co-author on this manuscript. According to our Data Policy, data must either be included with the submission, be placed in a public repository, or available from a third party contact point who must not be an author on the manuscript. Please revise your data statement to a non-author institutional point of contact, such as a data access or ethics committee, and send this to us via return email. Please also include contact information for the third party organization (this contact should be a central address and not a named person), and please include the full citation of where the data can be found.

**Response: Thank you for your valuable comments, which are very meaningful for us to further improve our manuscript. We have revised the data availability statement, stating that all our raw data are now stored in the public database Figshare**

5) Please ensure that the funders and grant numbers match between the Financial Disclosure field and the Funding Information tab in your submission form. Note that the funders must be provided in the same order in both places as well.

Currently, order of grants are different in both places.

**Response: Thank you for your valuable comments, which are very meaningful for us to further improve our manuscript. The funding sources and grant numbers have been aligned across all required sections in the submission system.**

**Response to reviewers**

**Response: First of all, I would like to thank the reviewers for their careful review and comments on our manuscript and for pointing out our shortcomings. These comments are very helpful to us. Next, I will respond to each of your comments in detail.**

**Reviewer#1：**

**1.** Please revise the text in general, as several formatting mistakes have been identified (spaces, no italic words, etc.).

**Response:** **Thank you for your valuable comments, which are very meaningful for us to further improve our manuscript. We have carefully checked the entire article and made corrections to any formatting errors.**

**2.** Enhance the quality of figures.

**Response:** **Thank you for your valuable comments, which are very meaningful for us to further improve our manuscript. We are also confused about the issue of graphic quality, because once exported to PDF files through the journal website, the image quality will be lost, but the images we uploaded when submitting the manuscript are all 600dpi or higher. After analysis, we believe that this may be because the images inserted into the manuscript are compressed by Word (we have fixed this problem).**

**3.** Figure 4, 5, and 6. Use an arrow or a line to indicate the damage.

**Response:** **Thank you for your valuable comments, which are very meaningful for us to further improve our manuscript. We have added red arrows to indicate lung damage.**

**4.** Line 221-231: What are the clinical symptoms that *A. baumannii* causes in mice?

**Response:** **Thank you for your valuable comments, *Acinetobacter baumannii* acute lung infection mice usually show shivering, erect hair, reduced activity, and decreased body temperature.**

**Original content: “**A BALB/C mouse model of acute pulmonary infection caused by *A.baumannii* was established by tracheal instillation of bacteria directly into the trachea….”

**Current content:** “A BALB/C mouse model of acute pulmonary infection caused by *A.baumannii* was established by tracheal instillation of bacteria directly into the trachea. Following bacterial infection, mice exhibited clinical signs including hypothermia, reduced activity, and ocular discharge.”——**L235-237**

**5.** Why didn’t you evaluate the virulence of the reisolated bacteria to determine if phage therapy reduces its virulence?

**Response:** **Thank you for your valuable suggestions. We will carry out relevant research in the future. In addition, as you mentioned, we did not re-isolate the bacteria to evaluate the effect of the phage on bacterial virulence. We will answer from the following three aspects:**

**1. Research focus and experimental design: The main purpose of this study is to evaluate the therapeutic efficacy and safety of phage vB_AbaS_qsb1 in a mouse acute pneumonia model. We focus on the ability of phage to clear bacterial load, improve host survival, and changes in inflammatory response, rather than the long-term effects of phage on bacterial virulence. 2. Complexity of bacterial virulence: The virulence of bacteria is affected by many factors, including gene mutation, environmental adaptation, etc. Even if the isolated bacteria show phenotypic changes, it is difficult to directly attribute them to phage treatment. Therefore, evaluating the effect of phage only by the virulence changes of re-isolated strains may be biased.**

**3. Future research prospects: Although this study did not directly evaluate the virulence changes of re-isolated strains after phage treatment, in the future, we can combine genome sequencing, phenotypic analysis and other methods to further explore the potential effects of Phage on bacterial virulence factors to more fully understand its therapeutic mechanism.**

**6.** Additionally, why didn’t you evaluate the virulence of phage-resistant colonies of *A. baumannii*?

**Response:** **Based on the same considerations as above, we did not further evaluate the toxicity of phage-resistant colonies.**

**7.** Line 78: in vitro must be in italic format.

**Response:** **Thank you for your valuable comments, which are very meaningful for us to further improve our manuscript. We have fixed this issue.**

**Original content: “In vitro studies have demonstrated…”**

**Current content: “*In vitro* studies have demonstrated…”——Line 78**

**8.** Line 86: space after point “].In this…”

**Response:** **Thank you for your valuable comments, which are very meaningful for us to further improve our manuscript. We have fixed this issue.**

**Original content: “enteritis[22].In this study…”**

**Current content: “enteritis[22]. In this study…”——Line86**

**9.** Line 94: again space after point.

**Response:** **Thank you for your valuable comments, which are very meaningful for us to further improve our manuscript. We have fixed this issue.**

**Original content: “.The phage”**

**Current content: “. The phage has”——Line92**

**10.** Line 97: in vivo must be in italic format.

**Response:** **Thank you for your valuable comments, which are very meaningful for us to further improve our manuscript. We have fixed this issue.**

**Original content: “**vB_AbaS_qsb1 in vivo. This**”**

**Current content: “**vB_AbaS_qsb1 i*n vivo*. This model**”——Line95**

**11.** Line 106: preprepared?

**Response:** **Thank you for your valuable comments, which are very meaningful for us to further improve our manuscript. We have corrected this erroneous statement in our resubmitted manuscript.**

**Original content: “**Following preprepared double-layer agar plates seeded with qsb1 and *ioag01*, and subsequent incubation at 37°C overnight**”**

**Current content: “**Prepare a double-layer plate by mixing qsb1 and *ioag01* evenly, and subsequent incubation at 37℃ overnight**”——Line 104**

**12.** Line 110: again space after point.

**Response:** **Thank you for your valuable comments, which are very meaningful for us to further improve our manuscript. We have fixed this issue.**

**Original content: “.Transmission electron microscopy”**

**Current content: “. Transmission electron microscopy”——Line 134**

**13.** Line 121: again space between phase(about…

**Response:** **Thank you for your valuable comments, which are very meaningful for us to further improve our manuscript. We have fixed this issue.** **In addition, we have made appropriate modifications to the content here as requested by another reviewer and in line with your suggestions.**

**Original content: “The one-step growth curve of qsb1 revealed a short latent period (about 20 minutes), followed by an exponential phase(about 100 minutes), with a burst size of approximately 23 plaque-forming units (PFU) /cell (Figure 1D)”**

**Current content: “We analyzed the one-step growth curve of qsb1 at an MOI of 0.01. The results showed an extremely short latent period, with host lysis occurring within 10 minutes, followed by a rapid release of progeny phages. The phage population entered a plateau phase at 100 minutes, reaching a burst size of approximately 69 plaque-forming units (PFU) /cell (Fig 1D).”——Line 117-121**

**14.** Line 122: again space after point.

**Response:** **Thank you for your valuable comments, which are very meaningful for us to further improve our manuscript. We have fixed this issue.**

**Original content: “**.Thermal stability analysis demonstrated…”

**Current content:** “. Thermal stability analysis demonstrated…”**——Line 121**

**15.** Line 331: Text says “Figure 5: High-dose phage treatment in mice with A.baumannii lung infection.” However, is figure 6 instead.

**Response: Thank you for your valuable comments, which are very meaningful for us to further improve our manuscript. We have corrected this erroneous statement in our resubmitted manuscript.**

**Original content: “Figure 5: High-dose phage treatment in mice with *A.baumannii* lung infection.”**

**Current content: “Fig 6: High-dose phage treatment in mice with *A.baumannii* lung infection.”——Line 348**

**Reviewer #2:**

**1.** L24 and in other places in the manuscript: it is unclear if these authors have contacted the ICTV to request the classification of their phage into a new genus. Therefore, they must be more cautious in the naming convention. Using the phrasing “…we propose to name Acinibactriovirus…” instead of “we named”. I strongly encourage the authors to contact the ICTV committee to formulate their proposition of a new genus.

**Response: Thank you for your suggestion. We appreciate your concern regarding the naming convention. We would like to clarify that we have followed the ICTV classification standards, as detailed in the publication “10.3390/v13030506”, “the Subcommittee has established 70% nucleotide identity of the full genome length as the cut-off for genera, calculated in the same way as the species cut-off”. Our results show that the ANI values of vB_AbaS_qsb1 and some species of the other two genera are 73.4% and 73.5%, respectively. However, there are also results showing that the ANI value of vB_AbaS_qsb1 and other phages of the two genera is "-1", which indicates that there is a large ANI difference between qsb1 and related phages.**

**Based on this, we have classified our phage into a new genus, and we have already submitted this proposal to the ICTV. The ICTV has informed us that they will review our proposal in the summer of this year . We have attached the ICTV’s reply letter (Additional Materials-1).**

**In light of this, we had modified the manuscript to use the phrasing “we propose to name Acinibactriovirus…” instead of “we named” to be more cautious in the naming convention as per your suggestion.**

**Original content: “which we have named as *Acinibactriovirus* ”**

**Current content: “which we propose to name *Acinibactriovirus* ”——L24**

**2.** L37: replace unique by novel

**Response: Thank you for your valuable comments, which are very meaningful for us to further improve our manuscript.** **In the resubmitted manuscript, we have revised the manuscript according to your suggestions.——This sentence now appears at L37 in the revised manuscript.**

**Original content: “**a unique bacteriophage **”**

**Current content: “**a novel bacteriophage **”——L37**

**3.** L59: please define the term ICU

**Response: Thank you for your valuable comments, which are very meaningful for us to further improve our manuscript. We have used the Intensive Care Unit (ICU) to define the term ICU. ——This sentence now appears at L60 in the revised manuscript.**

**Original content: “**particularly in ICU settings”

**Current content: “**particularly in Intensive Care Unit (ICU) settings”**——L59**

**4.** L68-69: this sentence is unclear without the word “antibiotics” added after “inherent” and after “acquire”. Please rephrase

**Response:** **Thank you for your valuable comments, which are very meaningful for us to further improve our manuscript. We have rephrased the sentence based on your suggestion.**

**Original content: “**The high mortality rate of *A.baumannii* infections stems from its inherent resistance and ability to acquire resistance genes”

**Current content: “**The high mortality rate of *A.baumannii* infections stems from its intrinsic antibiotics resistance and its remarkable capacity to acquire antibiotics resistance genes from the environment.”——L67-69

**5.** L86-89: please simplify by making two sentences. 1) .. we isolated from sewage a novel… baumannii. 2) We characterized its biological properties and genomic features.

**Response:** **Thank you for your valuable comments, which are very meaningful for us to further improve our manuscript. We have rephrased the sentence based on your suggestion.——This sentence now appears at L91-L93 in the revised manuscript.**

**Original content: “**we isolated and systematically characterized a novel virulent phage, vB_AbaS_qsb1, targeting A.baumannii from environmental sewage, with its biological properties and genomic features comprehensively detailed”

**Current content: “**We isolated a novel phage of Acinetobacter baumannii from sewage. We characterized its biological properties and genomic features.”——L86-88

**6.** L93: you should not jump into conclusions before introducing data. The significant potential should be removed from this sentence.

**Response:** **Thank you for your valuable comments, which are very meaningful for us to further improve our manuscript. We have deleted the statements (with significant potential for therapeutic applications against multidrug-resistant *A.baumannii*) that involved errors.**

**Original content: “**This classification underscores the novelty of our work, as it introduces a new genus of phages with significant potential for therapeutic applications against multidrug-resistant *A.baumannii*.”

**Current content: “**This classification underscores the novelty of our work, as it introduces a new genus of phage.”——L91-92

**7.** L102: remove “a novo-“

**Response: Thank you for your valuable comments, which are very meaningful for us to further improve our manuscript. We have deleted words (a novo-).——This sentence now appears at L111 in the revised manuscript.**

**Original content: “**Purification and morphology of a novo-bacteriophage vB_AbaS_qsb1**”**

**Current content: “**Purification and morphology of bacteriophage vB_AbaS_qsb1**”**——L100

**8.** L104: Remove “potent”.

**Response: Thank you for your valuable comments, which are very meaningful for us to further improve our manuscript. We have deleted the word (potent).——This sentence now appears at L113 in the revised manuscript.**

**Original content: “**A potent *A.baumannii* phage, vB_AbaS_qsb1,”

**Current content: “**A *A.baumannii* phage, qsb1,”——L102

**9.** L104: You should NOT give a full name to this phage before describing the genome that supports the naming convention. Instead use qsb1 here and later mention that the full name is vB…. But that in the manuscript you’ll keep qsb1.

**Response:** **Thank you for your valuable comments, which are very meaningful for us to further improve our manuscript. It is indeed not appropriate to have a standard name for phages when the phages are not officially named. Therefore, we will delete "vB_AbaS_" and only keep qsb1.**

**Original content: “**A potent *A.baumannii* phage, vB_AbaS_qsb1,”

**Current content: “**A *A.baumannii* phage, qsb1,”——L102

**10.** L106: Please rephrase as “following preprepared” is quite an unusual term.

**Response:** **Thank you for your valuable comments, which are very meaningful for us to further improve our manuscript. We originally intended to express the word "following prepreared" as "preparing a double-layer plate by inoculating a mixture of phage and bacteria on a pre-prepared solid medium", but after you pointed out our problem, we also thought that such a statement might be ambiguous and more suitable to be reflected in the Method section. Therefore, we used another concise text in the manuscript.**

**Original content: “**Following preprepared double-layer agar plates seeded with qsb1 and *ioag01*, and subsequent incubation at 37°C overnight,”

**Current content: “**Prepare a double-layer plate by mixing qsb1 and *ioag01* evenly, and subsequent incubation at 37℃ overnight”——L104

**11.** L111: correct the typo to the word “dimensions”.

**Response:** **Thank you for your valuable comments, which are very meaningful for us to further improve our manuscript. We have fixed that typo. ——This sentence now appears at L119 in the revised manuscript.**

**Original content: “**phage’s head dimensiona were approximately 70×80 nm”

**Current content: “**phage’s head dimensions were approximately 70×80 nm”——L108

**12.** L116: typing GMDCC 64831-B1 in Google does not provide a way to access this resource. The genomic sequence of this phage should be deposited in Genbank or equivalent public database.

**Response: Thank you for your valuable comments, which are very meaningful for us to further improve our manuscript.** **GMDCC 64831-B1 is the number we obtained when we deposited the phage species in the Guangdong Microbial Culture Collection Center. In response to this issue, we will upload a strain preservation certificate issued by the Guangdong Microbial Culture Collection Center for your review (Additional Materials-2).** **In addition, we have uploaded the complete genome sequence of this phage to the Genbank and disclose our depository number (In the "note" line under the "source" column). The Genbank accession number: PV107591, Or you can click this link to preview the Genbank information directly** [**https://www.ncbi.nlm.nih.gov/nuccore/PV107591.1/**](https://www.ncbi.nlm.nih.gov/nuccore/PV107591.1/)**, Or view the image (Additional Materials-3). This information is also disclosed in the revised manuscript.**

**Original content: “**The NGS raw sequencing data have been deposited in the SRA databases, SRA data accession: PRJNA1170883;

**Current content: “**The NGS raw sequencing data have been deposited in the SRA databases, SRA data accession: PRJNA1170883; The complete genome sequence of the phage has been uploaded to the Bankit database of genbank, accession number: PV107591”——L179-181

**13.** L118 and figure 1C: this graph represents the total amount of particles produced under different starting conditions that are not detailed in the legend. The initial amount of phage should be indicated and the ratio between initial and the final amount of particles should be a better representation of the “replication efficiency”. The individual points of the three replicates should be indicated as in panel F. In panel E as well the three independent values should be presented.

**Response:** **Thank you for your valuable comments, which are very meaningful for us to further improve our manuscript. After careful consideration, we agree that the “definition of replication efficiency” you suggested is indeed appropriate for evaluating the replication efficiency of phages at different MOIs. Therefore, we have defined replication efficiency as the ratio of the final phage titer to the initial phage titer. Based on our calculations, we have determined that the optimal multiplicity of infection (MOI) for the phage is 0.01. Additionally, we have revised Figure 1 by providing more comprehensive image details and an improved figure legend in accordance with your suggestions.**

**1.Original content: “**The study of the biological characteristics of qsb1 showed that the optimal phage replication efficiency was achieved at an multiplicity of infection (MOI) of 0.1 (Figure 1C)”

**1.Current content: “**The study of the biological characteristics of qsb1 showed that the optimal phage replication efficiency was achieved at an multiplicity of infection (MOI) of 0.01 (Fig 1C)”——L115-116 and Fig 1C

**2.Original content: “(C)** MOI of vB_AbaS_qsb1, showing phage titer following infection of its host at various MOIs.”

**2.Current content: “(C)**MOI of vB_AbaS_qsb1, showing phage titer following infection of its host at various MOIs. MOI ranged from 0.01 to 100, with phage particle counts of 1×10^6^ PFU/mL to 1×10^10^ PFU/mL. Phage titers were measured in triplicate, and replication efficiency was calculated as the ratio of final to initial titers.”——L137-139 Legend

**14.** L121: the one-step growth has not been performed in the optimal conditions. Usually, after mixing phage and bacteria at a low MOI, the suspension is diluted to prevent re-infection of bacteria at the end of the first infection cycle. Here, the latent period, which should be represented by a flat line with a stable amount of phage over time is in fact characterized by an increasing amount of phages, which suggest that some bacteria are already producing new particles. It is therefore likely that the calculation of the burst size is compromised. Authors should also provide a reference to the protocol and calculation used to determine these parameters. Finally, in panel F, there is no mention in the legend of the statistical test used.

**Response:** **First of all, we sincerely appreciate your insightful and valuable suggestions, which have greatly contributed to improving our study.**

**In our previous experiments, we measured the growth curve of the phage at a higher MOI. However, at such a high MOI, an excessive number of phages may have affected the accuracy of the growth curve results. Based on your suggestion, we conducted additional experiments at a lower MOI (0.01). In these new experiments, we measured the phage titer every 5 minutes during the first 20 minutes. However, the results still did not allow us to clearly observe the latent period of the phage.**

**We believe there are two possible explanations for this observation. First, even after minimizing time-related errors during the procedure, it is possible that this phage inherently has a very short latent period. Second, we observed that at this MOI, the phage-bacteria mixture could rapidly clear 10 mL of culture within 2 hours, indicating its strong lytic capability against this bacterium. Our experience further supports the notion that this phage exhibits potent lytic activity. Additionally, we observed that the phage growth curve reached a plateau at approximately 100 minutes, with a calculated burst size of approximately 69 PFU/cell.**

**1.Original content: “**At this MOI, phage proliferation exhibited a two-log increase within 6 hours under these conditions. The one-step growth curve of qsb1 revealed a short latent period (about 20 minutes), followed by an exponential phase(about 100 minutes), with a burst size of approximately 23 plaque-forming units (PFU) /cell (Figure 1D).”

**1.Current content: “**At this MOI, phage proliferation exhibited a two-log increase within 6 hours under these conditions. We analyzed the one-step growth curve of qsb1 at an MOI of 0.01. The results showed an extremely short latent period, with host lysis occurring within 10 minutes, followed by a rapid release of progeny phages. The phage population entered a plateau phase at 100 minutes, reaching a burst size of approximately 69 plaque-forming units (PFU) /cell (Fig 1D).——L116-121 and Fig 1D

**2. Original content: “(D)** One-step growth curve of vB_AbaS_qsb1.”

**2. Current content: “(D)** One-step growth curve of vB_AbaS_qsb1 at an MOI of 0.01. The initial bacterial concentration was 6×10^8^ CFU/mL, and the phage titer reached 4.2×10^10^ PFU/mL at the plateau phase. The burst size was calculated as the ratio of the plateau phage titer to the initial bacterial count. Data represent the mean of three independent experiments.”——L140-143 Legend

**In response to your request, we have revised the manuscript accordingly, including a detailed description of our protocol for calculating burst size in the figure legend. Lastly, we have also incorporated the statistical method used in Figure F, as per your recommendation.**

**Thank you once again for your thoughtful feedback, which has helped us enhance the clarity and rigor of our study.**

**1.Original content: “(F)** pH stability of vB_AbaS_qsb1. All samples were analyzed in triplicate, and the results are expressed as mean values ±SD.

**1.Current content: “(F)** pH stability of vB_AbaS_qsb1. All samples were analyzed in triplicate, and the results are expressed as mean values ±SD. The data were analyzed using two-way ANOVA. *****p* < 0.0001, ****p* < 0.001, ***p* < 0.01, **p* < 0.05.”——L148-149 Legend

**15.** In the legend of Fig S2, replace “nuclear” by “nucleic acid”

**Response:** **Thank you for your valuable comments, which are very meaningful for us to further improve our manuscript. We have revised the information in the legend of Figure S2 in the supplementary file, and listed the legend information of all supplementary files at the end of this manuscript as required by the journal.**

**Original content: “**Validation of the nuclear type of vB_AbaS_qsb1”

**Current content: “**Validation of the nucleic acid type of vB_AbaS_qsb1”——Figure S2

**16.** L150-152: since 95 CDS were detected and that 42 have a known function, then why only 51 instead of 53 have no function (replace role by function)?

**Response:** **Thank you for your valuable comments. We detected a total of 95 CDSs in the genome of the phage, of which 42 had known functions, and of the other 53 CDSs, 2 were tRNAs and the other 51 had unknown functions. It may be that we did not clearly state the existence of the two tRNAs, so we added this information in the manuscript.**

**Original content:** “Among these, 42 CDSs were predicted to encode proteins with known functions, while the remaining 51 were classified as hypothetical proteins with uncharacterized roles.”

**Current content:** “Among these, 42 CDSs were predicted to encode proteins with known functions, while the 51 were classified as hypothetical proteins with uncharacterized roles, and an additional 2 CDSs encoded tRNAs.”——L159-160

**17.** L177-188: this paragraph should be revised as the data provided on figure 3A is quite unusual. How could it be possible that isolate 224 and TRS1 cluster together with qsb1 while they barely share 2 and 20 % similarity. Then how the authors decided to next focus on Vieuvirus… this is clearly unclear!

**Response: Thank you for your thoughtful comment regarding the phylogenetic analysis presented in Figure 3A. After careful consideration, we believe that clustering qsb1 with TRS1 and isolate_224 into the same branch may be due to the algorithm forcibly grouping species that are very different from other species into one category, which means that these three phages may be quite different from other phages. In addition, for phages of the Vieuvirus and Obolenskvirus genus, we did not observe that phages and qsb1 were clustered together in the cluster analysis. At the same time, in the following study, we further explained that qsb1 had significant differences in both ANI and AAI values. Therefore, we believe that qsb1 is a phage independent of these two genera.**

**Original content:** “We compared all previously reported complete *Acinetobacter* phage genome sequences (74 in total) and noted that the remaining 14 phages with partial genomes displayed coverage rates ranging from 0% to 49% (Supplementary Table S2). This analysis revealed that vB_AbaS_qsb1 is closely related to *Acinetobacter* phage vB_AbaS_TRS1and MAG_*Caudoviricetessp* _Isolate_224 (Figure 3A), with both clustering in the same cluster, and indicating genus-level consistency. However, the results of collinearity comparison showed that the genomes of vB_AbaS_qsb1, vB_AbaS_TRS1 and Isolate_224 were extremely different (Figure 3B), and the coverage of vB_AbaS_qsb1 on vB_AbaS_TRS1 and Isolate_224 was extremely low (only 20% and 2%, respectively). Furthermore, NCBI records indicate that neither VB_AbaS_TRS1 nor Isolate_224 has been formally classified within a recognized genus. Therefore, we suggested that vB_AbaS_qsb1 is also a phage independent of any known genus.”

**Current content:** “We compared all previously reported complete *Acinetobacter* phage genome sequences (74 in total) and noted that the remaining 14 phages with partial genomes displayed coverage rates ranging from 0% to 49% (Supplementary Table S2).The clustering results (Fig 3A) showed that qsb1 was not clustered with the *Vieuvirus* and *Obolenskvirus* genus*,* which suggests that qsb1 may be significantly different from the phages in these genus. This analysis revealed that vB_AbaS_qsb1 is closely related to *Acinetobacter* phage vB_AbaS_TRS1 and MAG_*Caudoviricetessp* _Isolate_224 (Fig 3A), with both clustering in the same cluster, and indicating genus-level consistency. However, the results of colinearity comparison showed that the genomes of vB_AbaS_qsb1, vB_AbaS_TRS1 and Isolate_224 were extremely different (Fig 3B), and the coverage of vB_AbaS_qsb1 on vB_AbaS_TRS1 and Isolate_224 was extremely low (only 20% and 2%, respectively). The reason for this discrepancy is that the algorithm may classify these individuals that are very different from other species into the same branch (in fact, they have little similarity to each other).Therefore, we suggested that vB_AbaS_qsb1 is a phage independent of any known genus.”——L186-200

**18.** L201: it is now more clear on which basis the authors suggest to name a new genus.

**Response:** **Thank you for your valuable comments, which are very meaningful for us to further improve our manuscript. We have identified our phages according to the relevant classification criteria of ICTV and have submitted our proposal to ICTV.**

**19.** L223: here you should mention that your mice are neutropenic as they received cyclophosphamide, as mentioned in the method section. Therefore, it is unclear if the authors have evaluated phage safety on neutropenic mice?

**Response:** **Thank you for your valuable comments, which are very meaningful for us to further improve our manuscript. Our study evaluated the safety of phages following cyclophosphamide injection. Specifically, we assessed phage safety in mice after the induction of neutropenia, as described in the Methods section. The misunderstanding may have arisen due to a lack of clarity in our manuscript. To address this, we have revised the legend of Figure 4 to explicitly state that the phage group was evaluated for safety following cyclophosphamide injection. As demonstrated in our results, phages were found to be safe for mice after the induction of neutropenia.——L257**

**Original content:** “Figure 4: Mouse model of *A.baumannii* lung infection and phage safety evaluation….”

**Current content:** “Fig 4: Mouse model of *A.baumannii* lung infection and phage safety evaluation. Mice were injected intraperitoneally with cyclophosphamide (CTX)….”——L260

**20.** In figure 4, panel A, the group of phage treated is invisible. I also suggest to replace “phage treated” by “phage”. The word treated is implying that mice may be infected.

**Response:** **Thank you for your valuable comments, which are very meaningful for us to further improve our manuscript. In the survival curve, since no mice died in the phage group, 10^6 CFU bacterial treatment group, and control group within 7 days, the survival curves of the three groups overlapped when plotted (all had 100% survival rate for 7 days). To solve this problem, we added a significance analysis indicator to the row with 100% survival rate.** **In addition, as you pointed out, we corrected "Phage-treated" to "Phage" in the phage safety experiment.**

**Original content:** “Phage treated group”

**Current content:** “Phage group”—— Legend of Fig4 A and B and C

**21.** Panel B, the individual 5 values should be indicated for WBC and neutrophils counts. The associated methods should be in the main manuscript and not in the supplement.

**Response:** **Thank you for your valuable comments, which are very meaningful for us to further improve our manuscript. We have added the requested data scatter plots in Figure 4B and added the relevant methods in the Methods section of the manuscript.**

**Original content: “**None method”

**Current content: “**Complete blood count：After collecting whole blood from the mice, blood parameters were analyzed within 1 hour using an automated blood cell counter (XN-550,Sysmex America).”——Method：L617-619

**22.** Panel C: it seems that authors have included technical replicates, which is fine as long as it is mentioned in the legend.L617-619

**Response:** **Thank you for your valuable comments, which are very meaningful for us to further improve our manuscript. We have included the information about the technical replicates in Panel C in the figure legend.**

**Original content:** “**(C)** Relative levels of inflammatory cytokines (IL-1β, IL-6, and TNF-α) in mouse lung tissues (n=5)….”

**Current content:** “**(C)** Relative levels of inflammatory cytokines (IL-1β, IL-6, and TNF-α) in mouse lung tissues (n=5). Each experimental group included 5 mice, with each mouse undergoing triplicate measurements….”

**23.** L265-267: Please add the infection dose of bacteria (… infected mice (3x10^8 CFU) as well as the phage dose corresponding to the low/medium/high to help the reader appreciate the results without looking for the legend of figure 5.

**Response:** **Thank you for your valuable comments, which are very meaningful for us to further improve our manuscript. We have added the bacterial infection dose and the phage dose corresponding to the different phage treatment groups in the corresponding positions.**

**Original content:** “ pneumonia-infected mice…, Mock-treated group…, Medium-dose…, Low-dose…, High-dose… ”

**Current content:** “ pneumonia-infected mice (3×10^8^ CFU/mouse)**,** Mock-treated group (saline), Medium-dose (3×10^8^ PFU), Low-dose (3×10^7^ PFU), High-dose (3×10^9^ PFU)”——L286-292
**24.** Panel B: why the values are not shown for the 3 other groups (medium/low and mock)?

**Response:** **Thank you for raising this important methodological concern. Regarding the absence of data for the medium/low-dose and mock groups in Panel B, this decision was based on the following scientifically rigorous considerations:**

**Mortality-Driven Sample Limitation: By day 7 post-infection, we observed 87.5% mortality in both medium/low-dose groups, leaving only 1 surviving animal in each of these groups. According to the ARRIVE guidelines and statistical power analysis recommendations, a sample size of n=1 is insufficient to draw valid biological conclusions or perform meaningful statistical comparisons.**

**Focus on Therapeutic Efficacy: The preserved high-dose group (80% survival) versus control group comparison provides statistically robust data to demonstrate treatment efficacy.**

**25.** L307-329: I suggest the authors to reshuffle the organization of the data by presenting the dat from panels ACDBEF.

**Response:** **Thank you for your valuable suggestion. We agree that aligning the panel order in the figure with the order of presentation in the manuscript would improve clarity. We will reorganize the data in the figure to present panels A, B, C, D, E, F in the suggested sequence to ensure consistency with the manuscript structure.——Figure 6：L370**

**26.** L307-309: I disagree with the term “continuous rise” as the main rise is observed between 12 and 24h and that almost no rise is observed between 24 and 48h. The second graph on CRP in Panel B is not commented and the legend is inappropriate.

**Response:** **Thank you for your valuable comments, which are very meaningful for us to further improve our manuscript. according to your suggestion, we have modified the inappropriate expressions and rephrased the text. In addition, we have also corrected the unclear annotation and inappropriate legend of Figure 6B.**

**Original content:** “CBC results (Figure 6B) indicated a continuous rise in white blood cell counts over the first 48 hours, suggesting the progression of inflammation.”

**Current content:** “CBC analysis (Fig 6B) showed that phage treatment effectively suppressed infection-induced inflammation, as evidenced by lower WBC and C-reactive protein (CRP) levels compared to the mock-treated group, indicating reduced systemic immune activation.”——L329-332

**27.** L319-320: this sentence is unclear and should be rephrased

**Response:** **Thank you for your valuable comments, which are very meaningful for us to further improve our manuscript. We have reorganized the language to describe it.**

**Original content:** “Over time, the phage titer steadily increased, corresponding with bacterial colonization, as the phages continued to infect and alleviate infection symptoms.”

**Current content:** “Over time, the phage titer in the lungs of mice in the phage treatment group gradually increased, which was closely associated with the continuous decline in bacterial colonization in the lungs of these mice.”——L343-345
**28.** L341: please indicate how many independent experiments were pooled, or are authors referring to independent groups for each time point ? It these experiments are really independent, then they should have 3 independent groups of mock-treated, which does not seem to be the case. Please clarify.

**Response: Thank you for your valuable comments, which are very meaningful for us to further improve our manuscript.** **We appreciate the reviewer's question regarding the independence of our experiments and the number of replicates. In our study, we aimed to assess bacterial colonization in the lungs of mice at specific time points. For each time point, we used two experimental groups, with 6 mice per group. The bacterial load in the lungs was measured once per group, resulting in 6 data points per group per time point.**

**To clarify, these 6 data points were derived from a single independent experiment, meaning that the data were not pooled from multiple independent experiments. While we did not perform additional independent replicates, we believe that the use of 6 mice per group provides a robust sample size for statistical analysis within a single experiment. This approach is commonly used in similar studies to assess bacterial colonization in animal models^[1]^.**

**We acknowledge that performing multiple independent experiments would further strengthen the reproducibility of our findings. However, due to we focused on ensuring sufficient sample size within each experiment to achieve statistical power. The consistency of our results across different time points and treatment groups supports the reliability of our data. In future studies, we will consider incorporating additional independent replicates to further validate our findings. We hope this clarification addresses the reviewer's concern and provides a clear explanation of our experimental design.**

**Reference:**[1] Ashworth, E.A., Wright, R.C.T., Shears, R.K. *et al.* Exploiting lung adaptation and phage steering to clear pan-resistant Pseudomonas aeruginosa infections in vivo. *Nat Commun* **15**, 1547 (2024). <https://doi.org/10.1038/s41467-024-45785-z>

**Original content:** “**(C)** Bacterial CFU) in the lungs of mice; each symbol represents an individual mouse, and lines represent the mean. Data represent pooled results from independent experiments, with n = 6 mice per group per time point, normalized to lung weight.”

**Current content:** “**(D)** Bacterial CFU in the lungs of mice; each symbol represents an individual mouse, and lines represent the mean. Results are combination of 1 independent experiment, n = 6 mice per group，normalized to lung weight.”——Legend L375-376

**29.** L350: the lines representing the mean are barely visible, make them thicker

**Response: Thank you for your valuable comments, which are very meaningful for us to further improve our manuscript. We have thickened the mean line to make it visible as requested.——**L368-371and Figure 6C

**30.** L374-375: this sentence does not add any value to the discussion

**Response:** **Thank you for your valuable comments, which are very meaningful for us to further improve our manuscript. We have deleted it.**

**Original conten: “**lifestyle prediction analyses confirmed that it exhibits characteristics of a virulent phage. This integrase gene offers an interesting avenue for further study in phage-host interactions and gene-editing applications.”

**Current content:** “…lifestyle prediction analyses confirmed that it exhibits characteristics of a virulent phage.”——L402-403

**31.** L382-384: this is a redundant statement, please remove it.

**Response:** **Thank you for your valuable comments, which are very meaningful for us to further improve our manuscript. We have deleted it.**

**Original conten: “**genes that remain uncharacterized.Our findings thus establish vB_AbaS_qsb1 as the prototype of the newly proposed *Acinibactriovirus* genus, highlighting its distinct genomic features and promising therapeutic potential.”

**Current content:** “genes that remain uncharacterized….”——L409

**32.** L387: the term “stable inflammatory markers” is confusing, please consider using “no increase of inflammatory marker level”.

**Response:** **Thank you for your valuable comments, which are very meaningful for us to further improve our manuscript. We have modified this sentence as you requested.**

**Original content:** “with stable inflammatory markers and no significant lung tissue damage.”

**Current content:** “with no increase of inflammatory marker level and no significant lung tissue damage.”——L412

**33.** L398: the authors focused on the data from fig 6 to state that survival is about 50%. However, I propose them to critically re-assess their survival data by analyzing them at the same time point, ie 60H. At this time point, in fig 4, the dose of 3x10^8 CFU leads to roughly 50% of mortality, while it rises to 80% in figure 5 and 100% in figure 6. It is clearly suggesting that either mice were more susceptible or the exact infection dose was strictly identical (not taking into consideration that tracheal instillation is quite challenging). Nevertheless, in figure 5 and 6, the mortality of the phage treated group (10^9 PFU) leads to 0% and 35%, respectively. By comparing the difference between mock vs phage-treated groups, in figure 5 the gain in survival is 80% and 65%, respectively, which means an average “gain in survival” of approx. 70%.
As a reviewer I’m not asking the authors to adhere to this suggestion but rather critically discuss their data that show that the initial conditions were not so reproducible, which are therefore blurring the precise evaluation of the efficacy of the phage treatment.

**Response:** **Thank you for your valuable comments, which are very meaningful for us to further improve our manuscript. We acknowledge the your's concern regarding the variability in mortality rates across different figures (Figures 4, 5, and 6) for the same bacterial dose (3x10^8 CFU). This variability likely reflects technical challenges associated with tracheal instillation, as well as potential differences in mouse susceptibility and bacterial virulence between experiments. Despite these variations, the survival benefit conferred by phage treatment (10^9 PFU) was consistently observed, with an average gain in survival of approximately 70% across different experiments. This suggests that the therapeutic effect of phages is robust, even in the face of experimental variability. We agree that the initial conditions were not perfectly reproducible, which may have blurred the precise evaluation of phage efficacy. However, the consistent survival benefit observed in phage-treated groups across different experiments supports the conclusion that phage therapy is effective in this model. In future studies, we will aim to standardize the infection protocol further and increase the number of replicates to enhance the reproducibility of our findings. We appreciate the reviewer's suggestion to critically discuss these points, and We also further critically analyze our data in the manuscript.**

**Original content:** “Additionally, the survival rate of treated mice, although improved, remained at 50%.”

**Current content:** “Additionally, There was some variability in the survival rate of mice at the endpoint observed in two independent experiments (80% and 50%, respectively), which suggests that the results are not completely reproducible. We speculate that this may be due to potential differences in mouse sensitivity and bacterial virulence between experiments. This also may be attributed to mechanical damage during phage administration or the empirical nature of current phage therapy protocols”——L422-428

**34.** L406-415: most of this paragraph is redundant with other elements, it could be easily streamlined.

**Response:** **Thank you for your valuable comments, which are very meaningful for us to further improve our manuscript. We've streamlined this.**

**Original content:** “In conclusion, we successfully isolated and characterized a novel *A.baumannii* virulent phage, vB_AbaS_qsb1. This phage exhibited potent lytic activity both in vitro and in vivo, demonstrating its ability to effectively eliminate *A.baumannii*, highlighting its potential as a promising therapeutic agent. Additionally, this phage exhibited a favorable safety profile in animal models, with treatment using qsb1 rescuing over 50% of mice with acute *A.baumannii* pneumonia. Notably, qsb1 maintained high phage titers in the lungs 7 days post-administration, which may also indicate that qsb1 has certain potential in the preventive treatment of *A.baumannii* infection. As antibiotic resistance continues to rise, phage therapy holds great promise as an alternative or adjunct to traditional antimicrobial treatments.”

**Current content:** “In conclusion, We identified and characterized a novel virulent *A.baumannii* phage, vB_AbaS_qsb1, demonstrating strong lytic activity in vitro and in vivo. It effectively reduced bacterial load, improved survival rates, and maintained high lung titers for seven days post-administration, suggesting potential preventive applications. The phage exhibited a favorable safety profile, with no adverse effects observed. Given the rising antibiotic resistance, qsb1 represents a promising therapeutic alternative or adjunct to traditional antimicrobial treatments.”——L434-440

**35.** L535: What do you mean by “section 2.1”?

**Response:** **Thank you for your valuable comments, which are very meaningful for us to further improve our manuscript. We apologize for this error, which may be due to the fact that we numbered the contents of the methods section in the previous manuscript and mistakenly retained this information. Now, we have corrected it!**

**Original content: “**The bacterial culture of *A.baumannii* was prepared as described in section 2.1,…”

**Current content: “**The bacterial culture of *A.baumannii* was prepared as described above,…”——L564

**36.** L539: replace “suspensions” by “lysates”

**Response:** **Thank you for your valuable comments, which are very meaningful for us to further improve our manuscript. We have replaced “suspensions” with “lysates” as per your suggestion, including in the subtitles.**

**1.Original content:** “Preparation of bacterial culture and phage suspension”

**1.Current content:** “Preparation of bacterial culture and phage lysates”——L563

**2.Original content: “**Bacteria were removed from the phage suspension using a 0.22 μm microporous membrane,…”

**2.Current content: “**Bacteria were removed from the phage lysates using a 0.22 μm microporous membrane,…”——L568

**37.** L549: please indicate the anesthesia procedure and details

**Response:** **Thank you for your valuable comments, which are very meaningful for us to further improve our manuscript. We have added the mouse anesthesia details to this paragraph.**

**Original content: “**The anesthetized mouse was positioned supine on an intubation platform,”

**Current content: “**The anesthetized mouse (Mice were anesthetized via intraperitoneal injection of 2.5% Avertin at a dosage of 375 mg/kg) was positioned supine on an intubation platform,”——L579

**38.** L555: CTX should be mentioned much earlier. Was any control or reference of the neutropenia achieved by this protocol?

**Response: Thank you for your valuable comments, which are very meaningful for us to further improve our manuscript.** **Yes, in our previous study, we found that after one cycle of cyclophosphamide injection in mice, the white blood cell count decreased significantly. In addition, we also referred to the methods for constructing related pneumonia models [2], which also used cyclophosphamide to construct a mouse model of neutropenia.**

**Reference:**[2] Li, S., Wei, B., Xu, L. *et al.* A novel *Saclayvirus Acinetobacter baumannii* phage genomic analysis and effectiveness in preventing pneumonia. *Appl Microbiol Biotechnol* 108, 428 (2024). https://doi.org/10.1007/s00253-024-13208-0

**39.** L612: the DOI link to the original data is unclear. It provides access to two tables, named Vertical force and Shear force, which are not related to this study. This should be fixed.

**Response:** **Thank you for your valuable comments, which are very meaningful for us to further improve our manuscript. The raw data information related to this study has been uploaded to the dataset figshare (https://figshare.com/), with the DOI number “10.6084/m9.figshare.27635490”** **Or you can click this link directly to enter our database “ https://figshare.com/articles/dataset/My_rawdata/27635490?file=52193141 ”. These raw data were uploaded to the dataset when the manuscript was first submitted, but it may be due to technical reasons that we forgot to set it to be publicly visible, resulting in the editors and reviewers being unable to see our raw data. This error has now been corrected, and the raw data can be browsed or downloaded online.——L657**
