# Supplementary material for: A novel genus of virulent phage targeting Acinetobacter baumannii: Efficacy and safety in a murine model of pulmonary infection
Source: PLoS Pathog. 2025 Jun 20;21(6):e1013268. doi: 10.1371/journal.ppat.1013268 (PMC12208414; doi:10.1371/journal.ppat.1013268)
Supplement: Attachment — Submitted filename: Response_to_Reviewers_auresp_2.docx [file ppat.1013268.s012.docx]

­­­PLOS Pathogens

Ref: PPATHOGENS-D-24-02438R1

A Novel Genus of Virulent Phage Targeting *Acinetobacter baumannii*: Efficacy and Safety in a Murine Model of Pulmonary Infection
Response letter to Reviewers’ comments

Dear Editors and reviewers:

Thank you very much for considering our manuscript to be published in **PLOS Pathogens**. We have revised our manuscript according to the reviewers’ comments. All revised contents have been highlighted by yellow color in the revised manuscript. Furthermore, the point-to-point responses have also been presented in this response letter.

We hope this revision will meet the high standard of PLOS Pathogens.

**Response to the Editors:**

1. The full species name should be used at the beginning of the abstract. For example, “Acinetobacter baumannii is a notable opportunistic…” should be used instead of “A. baumannii is a notable opportunistic…”.

**Response: Thank you for your valuable comments, which are very meaningful for us to further improve our manuscript.** **We have corrected the spelling errors in the article as you requested and have also checked the article for possible spelling errors.——L17**

**Original content:** A. baumannii is a notable opportunistic…

**Current content: *Acinetobacter baumannii* is a notable opportunistic…**

2. Line 57. “Acinetobacter baumannii is a Gram-negative…” instead of “A.baumannii is a Gram-negative..”

**Response: Thank you for your valuable comments, which are very meaningful for us to further improve our manuscript.** **We have modified the article according to your request.——L57**

**Original content:** A.baumannii is a Gram-negative …

**Current content: *Acinetobacter baumannii* is a Gram-negative …**

3. Lines 167 and 522. “PhageScope” instead of “phagescope”

**Response: Thank you for your valuable comments, which are very meaningful for us to further improve our manuscript. We have modified the article according to your request.**

**1# Original content:** Phagescope did not detect any virulence or antibiotic resistance genes…

**1# Current content: PhageScope did not detect any virulence or antibiotic resistance genes…——L167**

**2# Original content:** Phagescope

**2# Current content: PhageScope——L522**

4. Line 167. “CARD” should be used instead of "CRAD". Moreover, this database should be properly referenced in the Materials and Methods section.

**Response: Thank you for your valuable comments, which are very meaningful for us to further improve our manuscript. We have modified the article according to your request.** **In addition, we have also correctly cited CARD in the Materials and methods section.**

**1#Original content:** Comprehensive scans using CRAD…

**1#Current content: Comprehensive scans using CARD…——L167**

**2# Original content: “ CARD not quoted”**

**2# Current content:** CARD[37]**——L522**

5. In line 104 and other instances throughout the manuscript, the strain name should be presented in regular (non-italic) font.

**Response: Thank you for your valuable comments, which are very meaningful for us to further improve our manuscript.** **We have revised the entire article where the strain name format was incorrect.**

**Original content:** *ioag01…*

**Current content: ioag01…——L104, L132, L303, L434, L435, L444, L581, L593**

6. Spacing between words and after punctuation marks should be carefully reviewed. For example, “A. baumannii” should be used instead of “A.baumannii”.
   Line 530. “Acinetobacter” instead of “AcineAtobacter”.

**Response: Thank you for your valuable comments, which are very meaningful for us to further improve our manuscript.** **We have corrected the spelling error as you requested and checked the full text to ensure it meets the journal requirements.**

**Original content:** *AcineAtobacter* phages*…*

**Current content: *Acinetobacter* phages…——L530**

**Response to reviewers #3**

1. L59: "Intensive Care Unit" appears in a different font compared to the rest of the text.

**Response: Thank you for your valuable comments, which are very meaningful for us to further improve our manuscript.** **We have corrected the formatting error as you suggested and have reviewed the entire text.**

**Original content:** Intensive Care Unit*…*

**Current content:** Intensive Care Unit**…——L59**

2. L87: Acinetobacter baumannii is not italicized. Additionally, since the full name of the bacterium has already been mentioned earlier, it should be abbreviated as A. baumannii.

**Response: Thank you for your valuable comments, which are very meaningful for us to further improve our manuscript.** **We have corrected the spelling problem according to your suggestion and will check the full text.**

**Original content:** Acinetobacter baumannii from sewage.

**Current content:** *A.baumannii* from sewage.**——L87**

3. L167: The absence of virulence genes and ARGs in the genome is assessed using the CRAD and Phagescope databases. Is “CRAD” correct, or did the authors mean “CARD”? Moreover, this database should also be mentioned in the Methods section.

**Response: Yes, we are referring to the use of CARD for virulence gene identification. We apologize for the typo here due to our negligence, and we have corrected it and checked the whole text. In addition, we have also correctly cited and described the database in the Materials and methods section.**

**1#Original content:** Comprehensive scans using CRAD…

**1#Current content: Comprehensive scans using CARD…——L167**

**2# Original content: “ CARD not quoted”**

**2# Current content:** CARD[37]**——L522**
